# Build, do not dismantle: leveraging a differentiated service delivery approach for broader health impact amidst funding changes

**DOI:** 10.1002/jia2.26514

**Published:** 2025-07-07

**Authors:** Anna Grimsrud, Charles B. Holmes, Linda Sande

**Affiliations:** ^1^ HIV Programmes & Advocacy, IAS – International AIDS Society Cape Town South Africa; ^2^ Center for Innovation in Global Health Georgetown University Washington DC USA; ^3^ Health Economics and Epidemiology Research Office Faculty of Health Sciences University of the Witwatersrand Johannesburg South Africa

“*It is so easy to break down and destroy. The heroes are those who make peace and build*.”—Nelson Mandela

We received over 100 abstracts in response to this supplement's call for evidence to advance the scale‐up of differentiated service delivery (DSD) beyond HIV treatment. However, since January 2025, the global context for HIV service delivery has shifted dramatically.

A steep, sudden reduction in United States government funding has jeopardised HIV services in many high‐burden countries [1]. The closure of United States Agency for International Development (USAID) and termination of President's Emergency Plan for AIDS Relief (PEPFAR) programming delivered through USAID partners [2] marks more than a bureaucratic reshuffle—it signals the potential unravelling of critical components of the global HIV response.

While a State Department waiver was intended to provide clarification to allow for life‐saving humanitarian assistance, it failed to deliver, resulting in widespread disruption of HIV services, including life‐saving treatment [3]. The punative choices reflected in the waiver also reveal a fundamental shift in the scope of U.S. support going forward. The cessation of most pre‐exposure prophylaxis (PrEP) programmes (except for pregnant and breastfeeding women), removal of earmarked funding for key populations and orphans and vulnerable children, and the elimination of HIV survey, surveillance and community‐led monitoring activities underscore the magnitude of the shift. These changes threaten to dismantle the very structures built to make HIV care more efficient, client‐centred and resilient. Among them is DSD—an approach that has transformed HIV services and remains key to sustaining and expanding access amid shrinking resources.

Prior to 20 January 2025, DSD had been scaled and integrated into national guidance, especially in Eastern and Southern Africa. Data from the Coverage, Quality and Impact Network (CQUIN) network of 21 countries in Sub‐Saharan Africa show that by 2023, a median of 76% of people on antiretroviral therapy (ART) accessed treatment through a less‐intensive DSD model [4]. Multi‐month dispensing (MMD) of ART, which expanded during COVID‐19 [5], is an enabler of DSD. Scale‐up of MMD has continued, with 45% of people on ART supported by PEPFAR outside of South Africa, or 6.67 million people, receiving 6MMD in July−September 2023 (personal communication, Lauren Bailey). The potential savings from DSD include cost and resource savings from less frequent clinic visits, both for clients and the health system [6, 7], and can increase human resource capacity [8].

During COVID‐19, the World Health Organization (WHO) recommended DSD components to support uninterrupted access to services: MMD of ART, MMD and prescribing of PrEP, scaled provision of HIV self‐testing and ART distribution through community distribution points [9]. While these elements remain relevant, the current trend is moving in the wrong direction—ART refill durations are being shortened [10, 11], community distribution points are dismantled and group models phased out with reduced healthcare worker funding, especially for lay cadres.

This supplement is more timely and relevant than ever. DSD adapts care around client needs—a flexibility that is essential in an era of austerity. In the face of funding cuts, DSD provides not a fallback plan, but a forward‐looking strategy. Since its articulation nearly a decade ago [12], DSD has focused on client‐centredness, grounded in the assumption that those who are clinically stable and treatment literate can be seen less frequently and will seek care when needed. DSD aligns with self‐care principles and ensures that resources are available when intensified care is required.

Crucially, DSD is not HIV‐specific. It offers a scalable framework for managing other chronic conditions and co‐morbidities among people living with HIV [13]. In today's resource‐constrained environment, the principles of DSD—client‐centredness, efficiency, flexibility—are not just relevant, but essential. Rather than dismantling the DSD framework, we should build upon it to support an integrated HIV service delivery model for the future.

This supplement demonstrates how DSD can go beyond sustaining HIV treatment. It can be a foundation for integrated chronic care, a model for resilience in fragile systems and a mechanism to safeguard key populations from being left behind. DSD offers a way to keep people at the centre and to build health systems from the ground up—efficiently, equitably and sustainably. The supplement includes research across four key themes.

The first theme is on DSD for HIV treatment alongside the integration of other health needs. Articles in this section explore how DSD can be used to deliver integrated care—for example, combining HIV treatment with hypertension or diabetes management. Three papers—Kiggundu et al. [14], Hickey et al. [15] and Pascoe et al. [16]—highlight the successes of integrating hypertension and HIV care within DSD models. In Uganda, Kiggundu et al. randomised clinics to implement hypertension screening and treatment into their HIV DSD models [14]. There were large numbers of people living with HIV with undiagnosed hypertension, 85% of the 3164 people with HIV and hypertension were newly diagnosed in the study. Presenting a mixed‐methods study, integration was concluded to be feasible and adaptable facilitated by the availability of resources and synchronisation of HIV and hypertension visits. In South Africa, Pascoe et al. reviewed the already integrated DSD approach in the country assessing the alignment of medication visits and dispensing intervals for people living with HIV and hypertension from 18 public sector clinics across models of service delivery [16]. The results highlight high degrees of alignment with facility visits and medication pick‐ups, 94% and 95%, respectively, and no increase in visit burden for co‐morbid clients. In the SEARCH study from Kenya and Uganda, Hickey et al. present an alternative approach to leveraging existing Ministry of Health staff to integrate HIV and non‐communicable disease care [15]. They show how Ministry of Health community health workers can effectively deliver integrated HIV and hypertension services at the community level, with active linkages to nearby health facilities. The magnitude of the chronic disease risk among people living with HIV in South Africa was described by Sahu et al. in the Kwa‐Zulu Natal province [17]. Among those in a community‐based ART model, nearly a quarter of participants smoked (24%), had hypertension (23%) and half (50%) were obese. These data highlight the urgent need to address both the prevention and treatment of chronic conditions among people living with HIV. It is encouraging to see growing opportunities and the increasing feasibility of integrating HIV care alongside common co‐morbidities.

The second theme examines how the DSD approach is being applied to other chronic conditions, with two papers on DSD for tuberculosis (TB), drawing lessons from HIV service delivery informing design and scale‐up. In a discrete choice experiment (DCE), Strauss et al. found strong preferences for DSD among people with TB in the Eastern Cape province in South Africa, with three classes of preferences—community‐based, clinic‐based and group‐models [18]. In Ferroussier‐Davis et al., outcomes among people living with TB accessing care and treatment through different DSD models in Uganda are presented, demonstrating the feasibility of both facility‐ and community‐based DSD models beyond HIV [19].

The need and the practical potential of extending DSD models to often‐overlooked populations is the third theme. In Hicks et al., results from implementing a risk assessment tool to adapt care for adolescents and young adults living with HIV in Kenya demonstrates that low‐intensity models can be provided to adolescents and young adults living with HIV without additional loss to follow‐up or viral non‐suppression [20]. In the low HIV prevalence setting of Cambodia, Yam et al. highlight that a community ART delivery model implemented during COVID‐19 was cost‐effective in reducing the decline in physical health in people living with HIV [21]. Bothma et al. present experiences of healthcare workers delivering a DSD model to trans clients [22]. They demonstrate the need for tailored transgender services as transgender clients continue to face negative experiences when seeking care in standard service delivery facilities. This evidence is particularly crucial in the current climate of shifting funding priorities that represent a major threat to person‐centred services for key populations. Another DCE from Australia assessed preferences among gay, bisexual and other men who have sex with men and highlighted the diverse preferences for the delivery of sexual health and PrEP services [23].

In addition to the work of Ong et al., DSD for PrEP emerged as the final theme of this supplement and is also the focus of Musheke et al. and Owidi et al. [24, 25]. In Zambia, Musheke et al. highlight the extended reach of PrEP for adolescent girls and young women in Zambia with decentralisation of PrEP services [24]. Similarly, Owidi et al. present the perspectives of both clients and providers in a pharmacy‐based PrEP programme, another intervention adapting the “where” building block of service delivery to expand access to PrEP [25].

Beyond these themes, Fernández Villalobos et al. present the scale of global ART DSD implementation prior to COVID‐19 across 175 facilities, offering a rare and valuable insight into its scope [26]. This information is particularly relevant for decision‐makers, especially in the context of limited resources, where there is a continued need to decongest facilities and prioritise care for clients requiring the most provider attention. And finally, in a viewpoint, Wilkinson et al. summarise the need for differentiation at re‐engagement, and the policy work done in South Africa and Zimbabwe to develop re‐engagement algorithms [27]. In both examples, the algorithms are designed both to support those with increased clinical needs and to enable rapid access to less‐intensive DSD models to support sustained engagement.

As we navigate this new era in global health and HIV service delivery, several key questions remain:

- **Will countries embed DSD into their primary care strategies?** In some settings, such as Kenya and Uganda [28, 29, 30], integrating HIV into chronic care platforms is seen as an efficient solution in response to cuts in foreign health aid. But will the core elements of DSD – client‐centredness, the separation of clinical visits from refill‐only visits and access to MMD – be retained? Can successes in DSD for HIV influence the expanded use of DSD models for other chronic conditions addressed in primary care settings?
- **What is the future of out‐of‐facility DSD models and of community healthcare workers?** Out‐of‐facility models have been instrumental in improving treatment access and adherence, particularly in rural areas, where travel to clinics is a challenge especially when compounded by a short supply of chronic medications [31]. Community health workers have been vital in facilitating group models, as well as providing treatment literacy and psychosocial support. Yet, these outreach models and lay cadres have often relied on external funding, and as that diminishes, will governments prioritise and sustain these components of the HIV response?
- **Will DSD be a core element of PrEP delivery going forward?** The COVID‐19 pandemic accelerated a DSD approach to PrEP [32], with a more flexible, less medicalised and more user‐centred approach—including, and especially for key and vulnerable people. However, with uncertainty in the HIV prevention landscape, will advocates push for a DSD approach for PrEP to be continued?
- **Will DSD support the scale‐up of future innovations in HIV drug delivery?** Long‐acting HIV prevention and treatment products could reshape the HIV response. In addition, new co‐formulations and self‐injectable delivery mechanisms will be part of the future response. These innovations will only reach their potential if delivered in a way that puts people first [33]. Will DSD be part of this future?

In the face of a precipitous decline in U.S. funding and an uncertain global funding context, HIV services are being reshaped at an alarming speed. This supplement offers timely, practical evidence to support ministries of health and finance, implementers and advocates in rethinking how HIV services are delivered. Amid this upheaval, DSD—including beyond HIV treatment—remains one of our strongest tools to sustain the impact of HIV response and uphold the premise of people at the centre. In addition, enablers of DSD such as MMD are particularly important now, as they can be cost‐saving for clients and resource saving for the health systems [34] during a period of increased resource constraint. We must resist the impulse to retreat and instead build on the innovations already in place. Yet, without deliberate intention and sustained commitment, DSD risks becoming a casualty of the current funding crisis. It is not only worth protecting; it is essential to the future of the HIV response and part of the antidote. We cannot allow short‐term decisions to unravel years of progress. Now is the time to build—not dismantle.

## COMPETING INTERESTS

The authors declare no competing interests.

## AUTHORS’ CONTRIBUTIONS

AG wrote an initial outline of the Editorial and the first draft. CBH and LS reviewed and added content. All authors approved the final version of the manuscript.

## Data Availability

Data sharing is not applicable to this article as no datasets were generated or analysed during the current study.
